# Akaluc bioluminescence offers superior sensitivity to track in vivo glioma expansion

**DOI:** 10.1093/noajnl/vdaa134

**Published:** 2020-10-10

**Authors:** Dominique Bozec, Anirudh Sattiraju, Alexandros Bouras, Joe G Jesu Raj, Daniel Rivera, Yong Huang, Chrystian Junqueira Alves, Rut Tejero, Nadejda M Tsankova, Hongyan Zou, Constantinos Hadjipanayis, Roland H Friedel

**Affiliations:** 1 Department of Neurosurgery, Icahn School of Medicine at Mount Sinai, New York, New York, USA; 2 Nash Family Department of Neuroscience, Friedman Brain Institute, Icahn School of Medicine at Mount Sinai, New York, New York, USA; 3 Department of Pathology, Icahn School of Medicine at Mount Sinai, New York, New York, USA; 4 Brain Tumor Nanotechnology Laboratory, Tisch Cancer Institute at Mount Sinai, New York, New York, USA

**Keywords:** Akaluc, bioluminescence imaging (BLI), glioblastoma (GBM), glioma, luciferase

## Abstract

**Background:**

Longitudinal tracking of tumor growth using noninvasive bioluminescence imaging (BLI) is a key approach for studies of in vivo cancer models, with particular relevance for investigations of malignant gliomas in rodent intracranial transplant paradigms. Akaluciferase (Akaluc) is a new BLI system with higher signal strength than standard firefly luciferase (Fluc). Here, we establish Akaluc BLI as a sensitive method for in vivo tracking of glioma expansion.

**Methods:**

We engineered a lentiviral vector for expression of Akaluc in high-grade glioma cell lines, including patient-derived glioma stem cell (GSC) lines. Akaluc-expressing glioma cells were compared to matching cells expressing Fluc in both in vitro and in vivo BLI assays. We also conducted proof-of-principle BLI studies with intracranial transplant cohorts receiving chemoradiation therapy.

**Results:**

Akaluc-expressing glioma cells produced more than 10 times higher BLI signals than Fluc-expressing counterparts when examined in vitro, and more than 100-fold higher signals when compared to Fluc-expressing counterparts in intracranial transplant models in vivo. The high sensitivity of Akaluc permitted detection of intracranial glioma transplants starting as early as 4 h after implantation and with as little as 5000 transplanted cells. The sensitivity of the system allowed us to follow engraftment and expansion of intracranial transplants of GSC lines. Akaluc was also robust for sensitive detection of in vivo tumor regression after therapy and subsequent relapse.

**Conclusion:**

Akaluc BLI offers superior sensitivity for in vivo tracking of glioma in the intracranial transplant paradigm, facilitating sensitive approaches for the study of glioma growth and response to therapy.

**Key Points**

- Akaluc is a novel sensitive luciferase reporter with more than 100-fold higher sensitivity for in vivo tracking of brain cancer expansion in intracranial transplant models.
- The Akaluc bioluminescence imaging system is an important addition to the tool box for brain cancer research, allowing more sensitive studies of glioma expansion and therapy responses using in vivo models.

**Importance of the Study**

The high sensitivity of Akaluc bioluminescence is a significant improvement for noninvasive tracking of tumor expansion in preclinical cancer studies. It facilitates more sensitive in vivo tracking of brain cancer growth in intracranial transplant models. It can be expected that Akaluc BLI will also find wide application in other cancer model paradigms, including the detection of small incipient tumors and micro-metastasis.

High-grade gliomas, such as glioblastoma (GBM), the most common primary malignant brain cancer, are intensely studied due to poor prognosis and lack of durable therapeutic responses in patients.^1^ Current preclinical research efforts in GBM tumor biology and therapies rely in large part on the in vivo paradigm of intracranial orthotopic transplantation of GBM cells in rodents.

Bioluminescence imaging (BLI) is a commonly used noninvasive method for longitudinal tracking of intracranial GBM growth in rodent models.^2^ BLI with tumor cells expressing firefly luciferase (Fluc) yields signals that can be used to approximate the size and tumor burden of intracranial GBM.^3^ However, the limited sensitivity of Fluc BLI restricts its use mainly to tumors reaching significance sizes, usually with initial implantation of more than 10^5^ glioma cells.^3^

Recently, the Akaluc BLI system has been developed in an effort to improve in vivo sensitivity of BLI. First, the new firefly luciferase substrate Akalumine (“aka” means red in Japanese) was synthesized, which emits light in the near-infrared range, leading to better tissue penetration than lights of shorter wavelength such as blue or green.^4^ A directed evolution approach was then applied to firefly luciferase to mutate it into an enzyme that achieves maximal BLI brightness with Akalumine, leading to the enzyme Akaluc.^5^ The Akaluc BLI system can generate more than 10 times brighter signals than Fluc in vitro, and the difference is even higher for in vivo conditions (up to 100 times brighter), where tissue penetration is a major factor affecting signal detection.^5^

Here, we have established for the first time Akaluc BLI in human and mouse high-grade glioma cells, and we have demonstrated that Akaluc BLI is a more sensitive approach for longitudinal tracking of intracranial GBM expansion and therapy responses than standard Fluc BLI approaches.

## Materials and Methods

### Glioma Cell Lines

The human GBM cell line U87MG was obtained from the American Type Culture Collection, and the murine high-grade glioma cell line GL261 was obtained from the repository of the National Cancer Institute. U87MG and GL261 cells were cultured in DMEM media with 10% FBS and 1:100 Pen-Strep.

The human GBM stem cell (GSC) line SD3 was obtained from Dr. Santosh Kesari, John Wayne Cancer Institute and has been molecularly characterized as IDH-wild-type GBM of proneural transcriptional subtype^6^; the GSC line G16302 has been established in the laboratory of Dr. Tsankova at Mount Sinai and has been characterized as IDH-wild-type GBM of mesenchymal subtype.^7^ GSC lines were cultured in complete human neural stem cell media (Stemcell) with EGF and bFGF growth factors on laminin (Invitrogen) coated dishes, as described.^6^

### Generation of Viruses for Akaluc and Fluc Expression

The plasmid pcDNA-Venus-Akaluc^5^ was obtained from the RIKEN BioResource Center (#RDB15781) and used as a template for subsequent cloning steps. A plasmid pcDNA-Venus-Fluc was generated by replacing the Akaluc coding sequence with firefly luciferase (luc2), which was derived from the plasmid MSCV-Luciferase-PGK-hygro (Addgene #18782). The Venus-Akaluc and Venus-Fluc cDNA fragments were inserted by PCR TOPO cloning into the Gateway entry vector pENTR/D-TOPO (Invitrogen) and then transferred by Gateway LR reaction (Invitrogen) into the destination plasmids pLenti-PGK-Neo-DEST and pLenti-PGK-Puro-DEST, respectively.^8^ The final plasmids were deposited at Addgene.org as pLenti-Venus-Akaluc (neo) and pLenti-Venus-Fluc (puro) (Addgene #124701 and #140328).

Lentiviral particles were produced by transfection of HEK293T cells with pLenti plasmid, envelope plasmid pMD2.G, and packaging plasmid psPAX2 (Addgene #12259 and #12260; deposited by Didier Trono, EPFL Lausanne). Cell supernatants were collected at 2–3 days after transfection and viral particles were concentrated by ultracentrifugation. Retroviral MSCV-Luciferase viral particles were generated by the transfection of Phoenix producer cells with MSCV-Luciferase-PGK-hygro and collection from supernatant following standard protocols.^9^

### Transduction of Glioma Cells

For lenti-Venus-Akaluc (neo) or lenti-Venus-Fluc (puro) transduction, glioma cells were transduced with lentiviral particles and selected starting 48 h after transduction with the appropriate antibiotics: for neo selection, 600–1000 µg/ml G418 for U87MG and GL261 cells, or 150 µg/ml G418 for GSC lines; for puro selection, 1 µg/ml puromycin.

For retroviral MSCV-Fluc transduction of glioma cells (U87MG, GL261), cells were transduced with MSCV-Luciferase retroviral particles and subsequently selected with 200–500 µg/ml hygromycin.

### Fluorescence-Activated Cell Sorting

To additionally enrich for cell populations with high lenti-Venus expression, antibiotic selected cells were gated by fluorescence-activated cell sorting (FACS; BD FACSAria IIu). Cells were dissociated with Accutase and resuspended in FACS buffer (Hibernate-E low fluorescence [BrainBits] with 0.2% BSA and 20 μg/ml DNase I [Worthington]) and passed through a 40 μm mesh filter into round-bottom tubes (Falcon). An example of a FACS gating experiment for cells with high Venus expression is shown for the line GL261-Venus-Akaluc in Supplementary Figure S1.

### Growth Curves

To assess cell proliferation, we used either the colorimetric CellTiter 96 AQueous One (Promega #G3580) proliferation test with cells that were seeded into a 96-well plate at 2500 cells/well, or the Incucyte live-cell imaging and analysis system (Sartorius) to continuously measure cell confluence with cells seeded into a 24-well plate at 33 300 cells/well. The seeding cell density was the same for both methods (17 500 cells/cm^2^).

### Glioma Cell Implantation in Mice

All animal procedures were approved by the Institutional Animal Care Use Committee of Icahn School of Medicine at Mount Sinai. For implantation of glioma cells into the right striatum of mice, 6- to 8-week-old C57BL/6 mice (Charles River) were used for GL261 implantation and ICR-SCID mice (Taconic) for SD3 GSC implantation. Mice were anesthetized with isoflurane and restrained in a small-animal stereotaxic instrument (David Kopf Instruments). A scalp incision was made, followed by a small cranial opening with a 26-gauge needle tip, 2 mm right and 0.5 mm anterior of Bregma. A Hamilton syringe attached to the stereotaxic frame fitted with a 26-gauge removable needle was used to inoculate glioma cells 2 mm below the cortical surface. The cranial opening was sealed with bone wax to prevent the backflow of cells. The skin was then reapproximated by the placement of interrupted 4-0 Vicryl sutures.

### BLI Substrates

The Akaluc substrate Akalumine-HCl was purchased from Sigma-Aldrich (TokeOni, #808350) or from FUJIFILM Wako (#018-26703), and a working solution of 2.5 mg/ml was prepared in H_2_O. d-luciferin was purchased from PerkinElmer (#122799), and a stock solution (30 mg/ml) was prepared by dissolving 1 g of d-luciferin in 33.3 ml of DPBS, and a working solution of 15 mg/ml was used for in vivo injections.

### Bioluminescence Imaging

BLI was performed using the IVIS Spectrum device, and Living Image 4.3 software was used for image analysis (Perkin Elmer). For in vitro BLI, d-luciferin or Akalumine-HCl was added to cells before imaging at a concentration of 250 μM. Starting at 2–3 min after substrate addition, measurements were taken every 1–2 min. Conditions for image acquisition: exposure time: 2 (or 5) s; field of view: D; binning: small; f/stop = 1. Baseline correction was performed by subtracting the BLI values from the respective parental cell lines. For in vivo BLI, mice were intraperitoneally injected before measurements with 150 mg/kg d-luciferin^3^ or 25 mg/kg Akalumine-HCl (approximating 75 nmol/g, described previously as sufficient for maximal Akaluc in vivo signal^5^). Heads of mice were shaved before imaging. IVIS parameters were kept constant throughout the experimental series: exposure time: 1 min; field of view: E; binning: large; f/stop: 1. Images were acquired every 2 min, and the maximal signal was chosen for subsequent analysis.

### Histology

For histological analysis of intracranial tumors of GSC transduced with lenti-Venus-Akaluc, cryopreserved mouse brains were sectioned at 20 µm thickness and glioma cells were identified by Venus fluorescence. Tumor spread was also routinely confirmed by staining sections with DAPI (Invitrogen) and anti-Human Nuclear Antigen (HNA; Millipore MAB1281), as described.^6^

### Statistical Analysis

GraphPad Prism version 8.2.1 was used for Kaplan–Meier survival analyses of mouse cohorts (Gehan–Breslow–Wilcoxon test) and to assess statistical significance between in vitro and in vivo BLI cohorts (multiple *t*-tests analysis; Holm-Sidak method; alpha = 0.05).

## Results

### Engineering of Glioma Cells With Akaluc and Fluc Reporters

**Figure 1.**
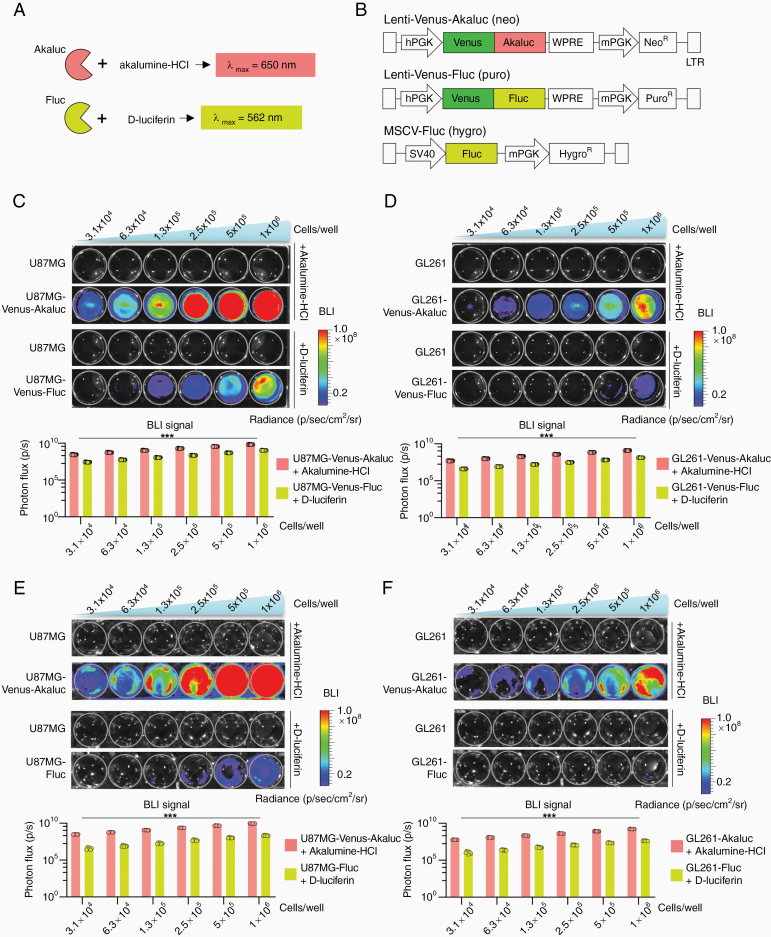
In vitro comparison of Akaluc and Fluc bioluminescence signals from glioma cells. (A) Principles of Akaluc and Fluc bioluminescence imaging (BLI): Akaluc BLI is based on the emission of light in the near-infrared spectrum by conversion of Akalumine-HCl substrate; Fluc BLI is based on emission in the green/yellow spectrum by conversion of d-luciferin substrate. (B) Diagrams of viral vectors to introduce luciferase expression in glioma cells. The lentivirus plasmids pLenti-Venus-Akaluc (neo) and pLenti-Venus-Fluc (puro) were generated for this study and have been deposited at Addgene (#124701 and #140328, respectively). The plasmid for MSCV-Fluc (hygro) was deposited by Scott Lowe at Addgene (#18782). (C and D) Images of luciferase BLI signals from U87MG (C) and GL261 (D) cells expressing Venus-Akaluc or Venus-Fluc (and parental lines as controls) over a range of cell densities in 24-well culture plates. Bar graphs below are based on 6 technical replicates for each condition, corrected for baseline. On average over the different densities, BLI signals emitted from U87MG-Venus-Akaluc cells were 9.8 times brighter than from U87MG-Venus-Fluc cells, and from GL261-Venus-Akaluc 11.2 times brighter than from GL261-Fluc. (E and F) Images of luciferase BLI signals from U87MG (E) and GL261 (F) cells expressing Venus-Akaluc or MSCV-Fluc (parental lines as controls). Bar graphs below are based on 6 technical replicates for each condition, corrected for baseline. On average, BLI emitted from U87MG-Venus-Akaluc was 48 times brighter than from U87MG-Fluc, and from GL261-Venus-Akaluc 38 times brighter than from GL261-Fluc. The BLI signals were significantly different between each pair of Akaluc versus Fluc cells at different cell densities (***adj. *P* value < .0001; multiple *t*-tests analysis, Holm-Sidak method, with alpha = 0.05).

For the initial validation of the Akaluc BLI system for glioma cells, we first conducted cell culture assays to compare side-by-side the sensitivity of the Akaluc and Fluc systems (Figure 1A). We transduced 2 commonly used glioma cell line models, the human GBM line U87MG and the murine high-grade glioma cell line GL261 with lentiviral vectors expressing either Venus-Akaluc or Venus-Fluc fusion proteins,^5^ or with a standard retroviral vector expressing Fluc alone (MSCV-Fluc; Figure 1B). Cell lines transduced with Lenti-Venus-Akaluc or Lenti-Venus-Fluc vectors were additionally gated by FACS to enrich for cells with high Venus expression levels. The transduction of glioma cells with viral vectors expressing Akaluc or Fluc luciferases had no significant effects on proliferation rates (Supplementary Figure S2).

### Akaluc-Expressing Glioma Cells Yield Higher BLI Signals In Vitro

We compared the sensitivity of Akaluc versus Fluc BLI in glioma cells plated into multi-well plates in serial dilutions. We first compared U87MG or GL261 cells transduced with either Lenti-Venus-Akaluc or Lenti-Venus-Fluc, and for both cell lines, we detected about 10-fold stronger BLI signals from the Akaluc-expressing cells than the Fluc-expressing cells (Figure 1C and D). We also conducted a comparison between Lenti-Venus-Akaluc and MSCV-Fluc expressing cells and obtained about 40- to 50-fold higher BLI signals from the former (Figure 1E and F), possibly reflecting the fact that for the MSCV-Fluc vector, enrichment for high expressor cells by FACS was not available due to absence of Venus or other fluorescent markers.

### More Than 100-Fold Higher BLI Signals From Akaluc-Expressing Glioma Cells in Intracranial Transplants

**Figure 2.**
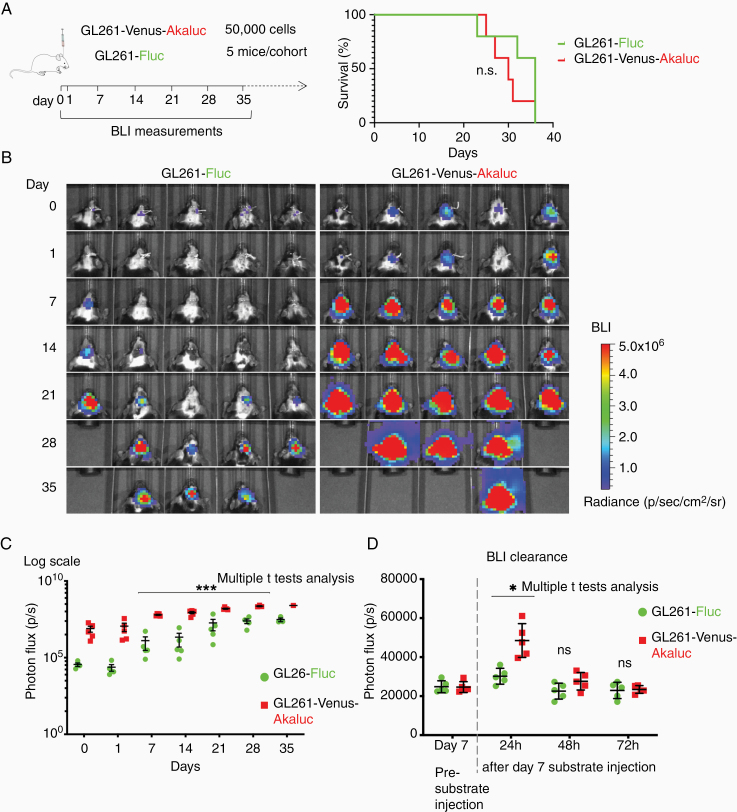
Higher sensitivity of Akaluc BLI for in vivo tracking of glioma expansion. (A) *Left*: Experimental timeline of mouse cohorts transplanted with GL261 glioma cells expressing either Venus-Akaluc or Fluc for longitudinal BLI measurements. *Right*: Kaplan–Meier survival analysis, which shows no significant survival difference between the 2 cohorts (n.s., not significant; *P* value = .27; Gehan–Breslow–Wilcoxon test). (B) Longitudinal BLI images of GL261-Venus-Akaluc and GL261-Fluc intracranial tumors demonstrate higher sensitivity of Akaluc over Fluc BLI for tracking in vivo tumor burden. (C) Quantitative comparison of BLI signals from the 2 cohorts of mice. The Akaluc BLI signals were on average about 220-fold higher than the Fluc signals on day 0, 480-fold higher on day 1, 48-fold higher on day 7, and the fold difference then declined with increasing size of the tumor, and reaching about 8-fold on day 35 (***adj. *P* value < .001; multiple *t*-tests analysis, Holm-Sidak method, with alpha = 0.05). On day 35, only one mouse of the GL261-Venus-Akaluc group and 3 mice of GL261-Fluc group remained alive. (D) BLI signals were measured before substrate injection on day 7 after transplantation, and the subsequent clearance of BLI signals was assessed by measuring at 24, 48, and 72 h after substrate injection. Note that BLI signals for Akaluc were still detectable after 24 h (*adj. *P* value = .01; multiple *t*-tests analysis, Holm-Sidak method, with alpha = 0.05), but returned to baseline after 48 or 72 h (ns, not significant).

We next examined the in vivo sensitivity of Akaluc versus Fluc BLI by intracranial engraftment of GL261-Venus-Akaluc and GL261-MSCV-Fluc glioma cells into syngeneic C57BL/6 mice. The better tissue penetration of the near-infrared light emitted by Akaluc BLI is predicted to confer a sensitivity advantage over Fluc in vivo. We recorded BLI signals from cohorts of 5 mice for each condition, starting shortly after implantation (~4 h) until 35 days post-implantation (Figure 2A).

Each mouse was transplanted with 50 000 GL261-Venus-Akaluc or -MSCV-Fluc cells, which is about 2- to 10-fold less than the number of cells typically used for intracranial xenografts.^3^ Before each imaging session, mice received intraperitoneal (i.p.) injection of the respective substrates at the recommended dosages (25 mg/kg Akalumine-HCl for Akaluc^5^ and 150 mg/kg d-luciferin for Fluc^3^). We observed no significant survival difference between the cohorts engineered for Akaluc or Fluc BLI, and all animals reached humane endpoints between 22 and 36 days post-transplantation (Figure 2A). We found that at all time points the BLI signals from the Venus-Akaluc cohort were significantly higher than those from the GL261-Fluc cohort (Figure 2B and C). The difference in sensitivity was particularly dramatic in the early phase of tumor growth, for example, about 480-fold on day 1, but the differences became less pronounced over time, possibly due to saturation effects or reduced substrate availability inside larger tumors. These data suggest that the high sensitivity of Akaluc is particularly useful for the detection of small tumors during the early stage of expansion in vivo. Our in vivo comparison here was conducted between GL261 Lenti-Venus-Akaluc and MSCV-Fluc cells. Taking into consideration that the in vitro data shown above demonstrated that in our hands Venus-Fluc produced higher BLI than the MSCV-Fluc (Figure 1), and that in vivo paradigms increase the advantage of Akaluc versus Fluc about 10-fold due to better tissue penetration, we extrapolate that Venus-Akaluc would be in in vivo intracranial transplant comparisons about 100 times more sensitive than Venus-Fluc.

We noted that some BLI signals from Akaluc remained detectable even 24 h after substrate injection, reflecting the high sensitivity of the system, while the BLI signals from Fluc had already returned to near undetectable levels by 24 h (Figure 2D). As a practical consequence, this implies that presubstrate injection images, which are commonly used for the setting of baseline values in BLI studies, should not be taken on the day following a previous Akaluc BLI experiment.

### High Sensitivity of Akaluc BLI to Track GSCs in Intracranial Xenografts

GSCs recapitulate the pathophysiology of original patient gliomas more faithfully than traditional GBM cell lines and display invasive growth behavior in intracranial transplants.^10^ We therefore tested Akaluc BLI in 2 patient-derived GSC lines for intracranial xenografts studies, G16302 (mesenchymal GBM subtype^7^) and SD3 (proneural subtype^6^).

**Figure 3.**
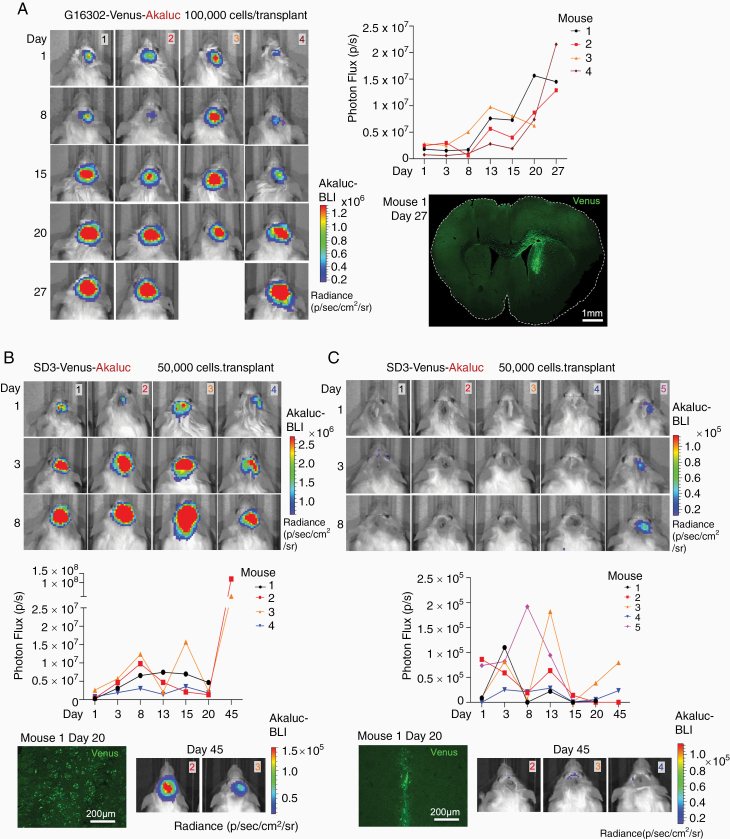
Akaluc BLI system enables sensitive in vivo tracking of GBM stem cell transplants. (A) *Left*: A cohort of mice implanted with 100 000 G16302-Venus-Akaluc GBM stem cells (GSCs) followed by BLI for up to 27 days after implantation (mouse 3 died on day 26). *Right-top*: Graphs show a progressive increase of BLI signals. *Right-bottom*: Fluorescence image of a coronal brain section from mouse 1 on day 27 after transplantation, showing the spread of G16302 tumor, visualized by Venus fluorescence. (B) *Top*: Akaluc BLI in a cohort of mice implanted with 50 000 SD3-Venus-Akaluc GSCs for up to 45 days. Graphs show BLI signals at each time point. *Bottom-left*: Fluorescence image of a coronal brain section from mouse 1 on day 20 after transplantation. Tumor cells are visible by Venus fluorescence. *Bottom-right*: Akaluc BLI detection in mouse 2 and mouse 3 on day 45 after transplantation. (C) *Top*: Akaluc BLI in a cohort of mice implanted with 5000 GSC-Akaluc cells SD3. *Bottom-left*: Fluorescence image of a coronal brain section from mouse 1 on day 20 after transplantation, showing clustering of Venus^+^ tumor cells along the implantation tract. *Bottom-right*: Akaluc BLI detection in mouse 2, mouse 3, and mouse 4 on day 45 after transplantation.

We first implanted 100 000 Akaluc-expressing G16302 GSCs into the striatum of adult SCID mice. We detected BLI signals starting from day 1 after transplantation in all animals, and the signals progressively increased up to day 27 (Figure 3A). Histological examination of mouse brains on day 27 revealed the establishment of invasive G16302 tumors, as indicated by Venus fluorescence (Figure 3A).

To investigate the benefit of using the Akaluc BLI system for the detection of smaller GSC tumors, we next implanted a cohort of SCID mice with only 50 000 Akaluc-expressing SD3 GSC into the striatum of each mouse, which is about 2- to 4-fold fewer cells than the number of cells typically used for intracranial transplants. Similar to G16302 tumors, we detected BLI signals on day 1 after transplantation in all animals, and the signals progressively increased in the ensuing 8 days (Figure 3B). Interestingly, the BLI signals then plateaued between day 8 and day 20 post-implantation, followed by a resurgence detected on day 45 (Figure 3B). This may indicate a prolonged period of engraftment of GSCs during the second and third weeks after implantation, during which time invading GSCs are engaged in extensive contact with the tumor microenvironment. Histological examination of tumor growth, as visualized by Venus fluorescence signals at 20 days post-implantation, showed diffuse invasion of tumor cells into surrounding brain parenchyma, confirming successful engraftment (Figure 3B).

The high sensitivity of the Akaluc BLI system should allow tracking of even smaller numbers of transplanted glioma cells, thus facilitating a more accurate modeling of tumor initiation and early stage of tumor growth. We therefore tested the detection threshold by measuring BLI signals from a cohort of mice transplanted intracranially with only 5000 Akaluc-expressing SD3 GSCs. We were able to detect low BLI signals on day 1 after transplantation, which slightly increased in the ensuing days, but then plateaued from day 3 to day 13 post-implantation (Figure 3C). The intensity of the BLI signals then showed a decrease after this time point until day 20, which may reflect increased tumor cell death from inefficient engraftment due to the small number of implanted GSCs. We followed 3 mice for longer time periods and were able to detect in 2 animals a rebound of BLI signals at 45 days, indicating subsequent successful tumor expansion (Figure 3C). Histological examination of one intracranial transplant on day 20 post-implantation indeed showed that at this time GSCs were still largely clustered near the injection tract, not yet established extensive tumor infiltration (Figure 3C). Taken together, the high sensitivity of the Akaluc BLI method allowed us to follow the early stage of tumor establishment and growth, which revealed a plateau period during tumor engraftment before the wider expansion of tumors.

### Akaluc BLI for Longitudinal Study of Therapeutic Effects With In Vivo Glioma Model

**Figure 4.**
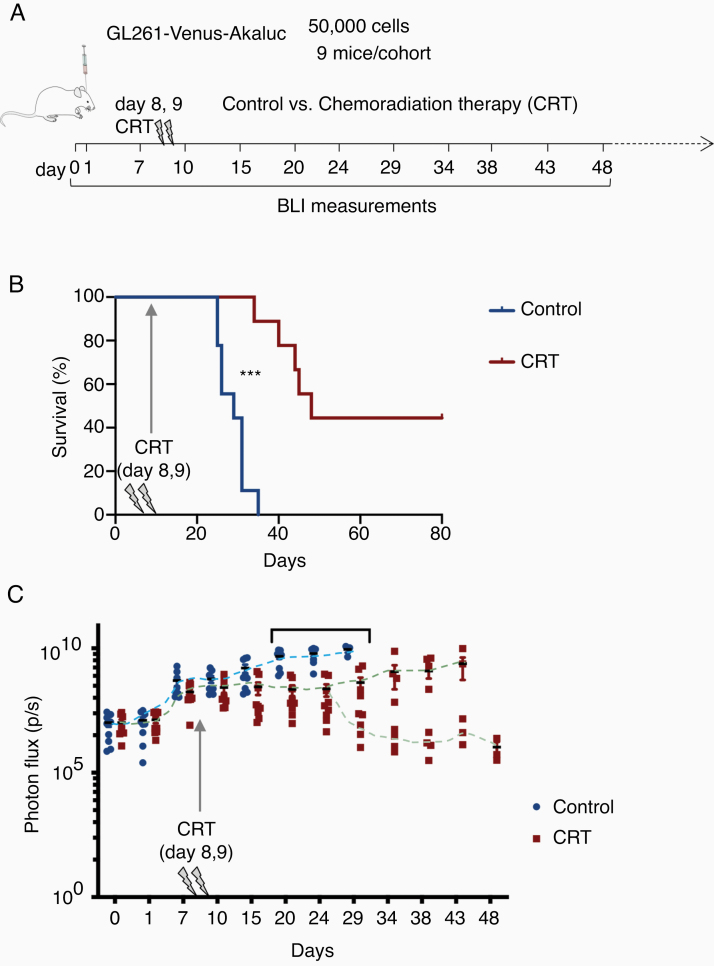
Akaluc BLI for in vivo tracking of therapy response of intracranial GBM transplants. (A) Experimental timeline of in vivo tracking of intracranial tumor burden by Akaluc BLI upon administration of chemoradiation therapy (CRT: Temozolomide [5 mg/kg] + X-ray [3 Gy] on days 8 and 9). (B) Kaplan–Meier survival curves show a significant survival difference in mice bearing GL261-Akaluc treated with CRT as compared with control (*n* = 9 mice per cohort; ***P = .0001; Gehan–Breslow–Wilcoxon test). (C) Changes in tumor burden measured by Akaluc BLI and quantified for treatment versus control cohorts. *P < .05; multiple *t*-test analysis. Dashed lines depict trajectories of tumor burdens. Note the bifurcation in the CRT cohort.

As a proof-of-concept to demonstrate the utility of the Akaluc BLI system for longitudinal tracking of glioma responses to therapy in vivo, we carried out chemoradiation therapy (CRT) on mice bearing GL261-Venus-Akaluc transplants. To approximate the standard-of-care therapy for GBM patients, we delivered partial fractionated irradiation of 3 Gy/session with concomitant temozolomide (5 mg/kg) on days 8 and 9 post-grafting (Figure 4A). Indeed, the survival of the treatment cohort was significantly improved compared to the control cohort, with median survival extended from 30 days in the control to 64 days in the CRT cohort (Figure 4B). At 80 days post-implantation, several mice were still alive in the CRT cohort, but none in the control cohort.

We followed the BLI signals in the 2 cohorts at regular intervals. In the control cohort, we observed a steady increase of the BLI signals corresponding to tumor expansion from day 1 post-implantation until the onset of tumor-related mortality beginning on day 24. In contrast, in the CRT cohort, we observed a plateau and even a slight decline of the BLI signals after CRT, indicating the effectiveness in slowing down tumor expansion. About 2 weeks after CRT treatment, we observed a bifurcation within the CRT cohort, where one group (5 of 9 mice) continued to display reduced BLI signals, indicating no tumor rebound, while the other group (4 of 9) showed a steady increase of BLI signals, indicating tumor re-expansion, which was associated with tumor-related mortality starting from day 35 in this group (Figure 4C). By the study endpoint of day 48, all the 4 mice showing tumor rebound died, whereas all 5 mice in the group showing no evidence of tumor rebound remained alive.

## Discussion

Sensitive noninvasive methods for longitudinal tracking of tumor growth kinetics are important tools to study in vivo tumor expansion and assess the effectiveness of therapy. Here, we demonstrate the utility of Akaluc BLI for in vivo tracking of tumor expansion in orthotopic GBM transplant models. Our data lead us to estimate more than 100-fold higher signal intensity from Akaluc BLI as compared to standard Fluc BLI for in vivo glioma tracking. The high sensitivity of Akaluc BLI enables tumor detection during the early stages of tumor establishment. It also permits an accurate assessment of therapy response and early detection of tumor recurrence.

The Akaluc BLI system was helpful for the detection of intracranial tumor growth in cases of low cell number transplant (eg, 5000 cells as compared to the typical 100 000 cells). We found that the sensitive Akaluc BLI method revealed that GSCs undergo a plateau phase after initial transplantation, perhaps reflecting a prolonged engraftment period before reinitiating tumor expansion around 20 days after transplantation. It is worth noting that GSCs display an infiltrative tumor growth pattern that is unique to GSC transplants,^10^ such that during the engraftment period, there are extensive interactions between invading GBM cells and tumor microenvironment, possibly resulting in significant tumor cell death. This is in contrast to serum grown glioma cell lines such as GL261, which typically expand as a continuous tumor mass.

One general limitation of the BLI method is that the correlation of BLI signals with the size of tumor decreases for very large tumors. For example, it has been reported that intracranial tumors larger than 40 mm^3^ start to plateau in their BLI signals, and small-animal MRI might be better suited to assess these larger tumors.^11^ We thus envisage that the advantages of Akaluc BLI are mainly relevant for studying the early stages of tumor growth or relapse.

A practical consideration for the adoption of Akaluc BLI for in vivo cancer studies is that Akalumine substrate is at present (2020) about 10 times more expensive than luciferin (based on recommended doses for in vivo studies). However, with the increased use of the Akaluc BLI system, the cost is expected to decline when larger amounts of the substrate are produced.

Of note, other efforts to improve the sensitivity of BLI for in vivo imaging include directed evolution of luciferase from marine organisms, which typically emit blue light, such as Nanoluc, for alternative substrates that emit light of longer wave lengths, or the coupling of blue-emitting BLI systems with orange fluorescent proteins for FRET effects.^12,13^ Such approaches may potentially reach in vivo sensitivity similar to that of Akaluc BLI and represent useful additions due to their distinct substrate specificities, thus allowing 2-population BLI.^13^

In summary, Akaluc BLI offers a sensitive method for in vivo tracking of glioma expansion, which is particularly useful for imaging of smaller tumors, as well as tumor relapse after therapy. We also believe that the Akaluc BLI model may also be instrumental for the study of low-grade gliomas in preclinical rodent models, which typically expand at a slower pace. It can also be predicted that Akaluc BLI will be highly useful for other in vivo models of cancer, including for detecting micro-metastasis.

## Supplementary Material

vdaa134_suppl_Supplementary_Data

## Notes

**Authorship statement**. Concept and design: R.H.F., C.H., and H.Z.; development of methodology: A.S., D.B., Y.H., C.JA., R.T., and N.M.T.; acquisition of data: D.B., A.S., A.B., J.G.J.R., and D.R; data analyses: A.S., D.B., R.H.F, H.Z., and C.H.; manuscript preparation: D.B., A.S., R.H.F., H.Z., and C.H.
