# Supplementary material for: Akaluc bioluminescence offers superior sensitivity to track in vivo glioma expansion
Source: Neurooncol Adv. 2020 Oct 10;2(1):vdaa134. doi: 10.1093/noajnl/vdaa134 (PMC7680182; doi:10.1093/noajnl/vdaa134)
Supplement: vdaa134_suppl_Supplementary_Data [file vdaa134_suppl_supplementary_data.pdf]

Supplementary Data

Akaluc bioluminescence offers superior sensitivity to track in vivo glioma expansion

Bozec D., Sattiraju A, et al.

Supplementary Figures S1 and S2.

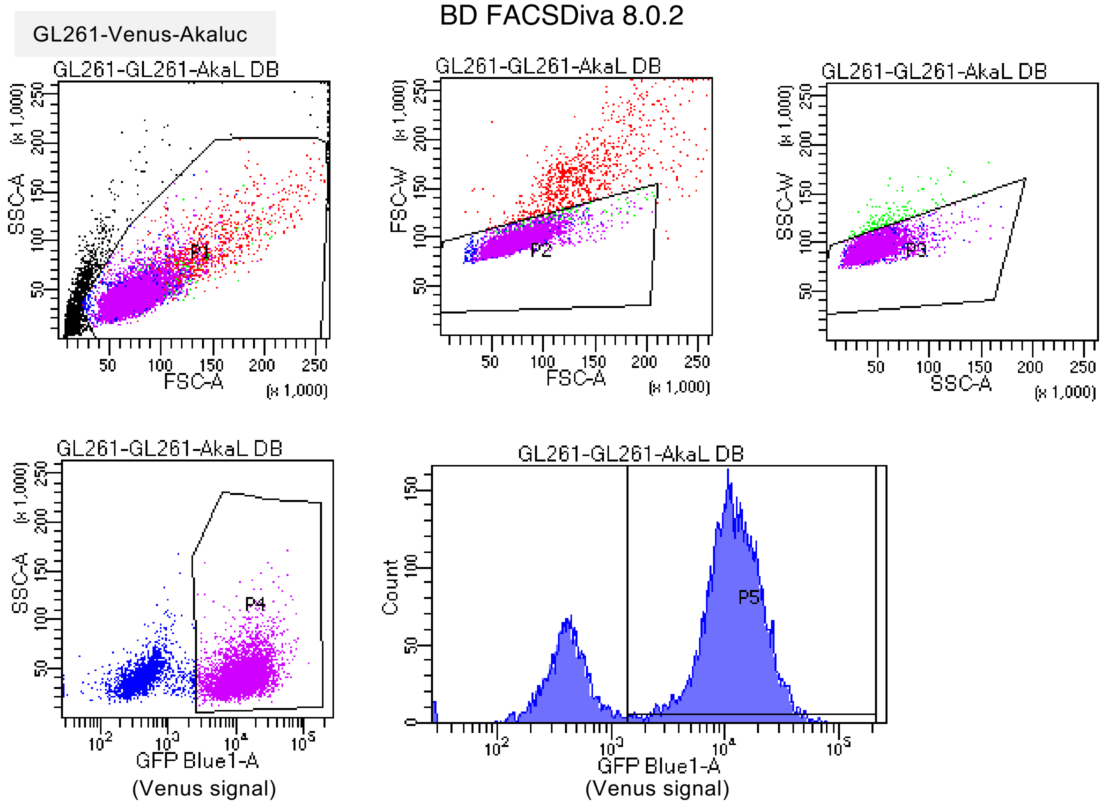

Figure S1. FACS gating of GL261-Venus-Akaluc cells based on Venus fluorescence.
To enrich transduced cell populations for high expression of lentiviral Venus-Akaluc, cells were gated by FACS for high Venus expression. Shown here is an example of FACS sorting results for GL261 that were transduced with Lenti-Venus-Akaluc vector and selected with G418 for more than one week. Cells were sorted through the gate P4, collected, and expanded for use in subsequent experiments.

Fig. S2

A

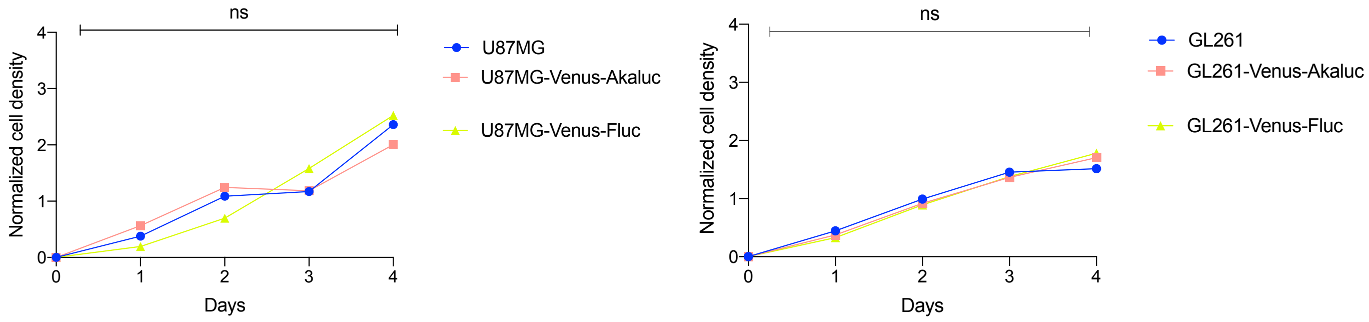

B

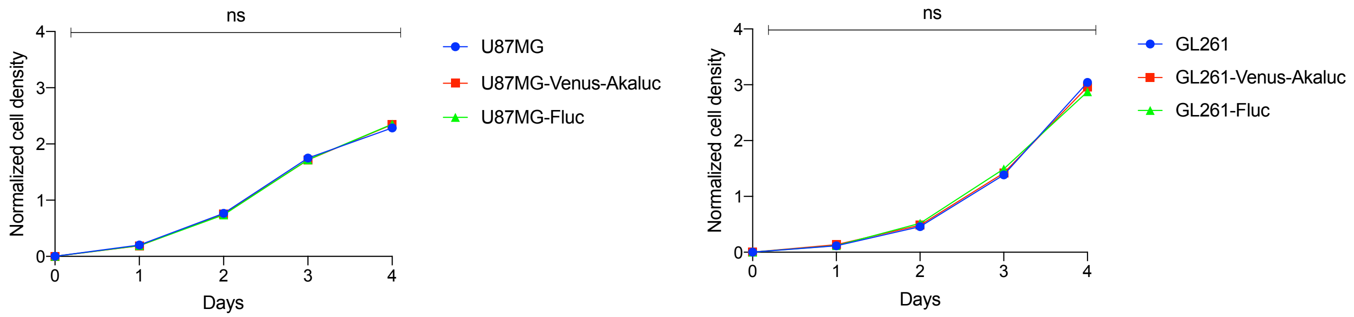

Figure S2. Growth curves of transduced GBM cells.

A) Comparison of cell growth curves of U87MG or GL261 glioma cells transduced with either Lenti-Venus-Akaluc or Lenti-Venus-Fluc vectors (un-transduced parental lines served as controls). Cells were seeded into 24-well plates and cell confluence was measured daily with Incucyte live imaging system.

B) Comparison of growth rates of U87MG or GL261 GBM cell lines transduced with either Lenti-Venus-Akaluc or MSCV-Fluc (un-transduced parental lines served as controls). Cell growth was determined by MTS colorimetric assay of cells seeded into 96-well plates.

No significant difference was detected between the cell lines in each experiment (ns, not significant; adj. p value > 0.99; one way ANOVA, Tukey's multiple comparisons test).
